# Predictors of Complications and One-Year Mortality in Adult Patients With Femoral Neck Fractures: A Retrospective Study

**DOI:** 10.7759/cureus.106212

**Published:** 2026-03-31

**Authors:** Ali H Alyami, Khalid A Alharbi, Razan S Alharbi, Sharifah Hussain Almasoud, Lamar Maddawi, Sana Alfahmi, Abdullah Alqthmi, Majd Saem Aldahar

**Affiliations:** 1 College of Medicine, King Saud Bin Abdulaziz University for Health Sciences, Jeddah, SAU; 2 College of Medicine, Umm Al-Qura University, Makkah, SAU; 3 Medicine, Qassim University, Qassim, SAU; 4 College of Medicine, Majmaah University, Majmaah, SAU; 5 Orthopedic Surgery, National Guard Hospital, Jeddah, SAU

**Keywords:** fracture femoral neck, hips, length of hospital stay (los), mortality, postoperative complication

## Abstract

Introduction

Femoral neck fractures (FNFs) are associated with significant morbidity and mortality, particularly in older adults. This study aimed to identify predictors of complications and mortality among patients with FNFs in a tertiary care center in Saudi Arabia.

Methods

A retrospective review was conducted of 183 patients admitted with FNFs between January 2016 and August 2024. Demographic, clinical, and surgical data were collected. Statistical analyses included descriptive summaries, univariate comparisons, and multivariate logistic regression to identify independent predictors of complications and mortality.

Results

The mean age was 71.8 ± 17.0 years, and 104 (56.8%) were female. Surgical treatment was performed in 171 (93.4%) patients, most commonly hemiarthroplasty 113 (61.7%). Complications occurred in 48 (26.2%) patients, while 13 (7.1%) patients died within one year. Patients with complications had significantly longer hospital stays (13.5 ± 12.8 vs. 9.6 ± 6.2 days, p = 0.007). Mortality was associated with the Garden classification (p = 0.011), chronic kidney disease (p = 0.039), conservative management (p = 0.013), and postoperative infection (p = 0.042). On multivariate analysis, the Garden classification independently predicted mortality (OR 3.09, 95% CI 1.10-8.67, p = 0.032).

Conclusion

Garden classification independently predicted mortality, while chronic kidney disease, postoperative infection, and conservative management were associated with mortality on univariate analysis. Complications significantly prolong hospitalization. To improve results, high-risk patients must be treated early, and perioperative care must be optimized.

## Introduction

In orthopedic surgery practice, femoral neck fractures are a common injury that carries a high morbidity and mortality rate [1]. Proximal femur fractures, which are frequently referred to as "hip fractures," include femoral neck fractures (FNFs) as a subgroup [2]. Moreover, with an incidence rate of 5 in 10,000 per year, femoral neck fractures account for nearly 3% of all hip fractures [3].

An expanding patient lifespan, degree of activity, and incidence of osteoporosis are thought to be some of the variables leading to the rising number of femoral neck fractures [4]. The classification of FNFs is important for understanding their nature and guiding appropriate interventions. FNFs are categorized using several methods, such as the Garden classification, which classifies FNFs using the degree of displacement [5,6]. Hip and pelvic radiographs, together with clinical findings, are used to make the diagnosis. The selected treatment plan is influenced by the type of fracture (with or without displacement in coxa vara, coxa valga), the patient's age, level of autonomy, and comorbidities [7]. Selected treatment options include hemiarthroplasty (HA), total hip arthroplasty (THA), osteosynthesis like open reduction and internal fixation (ORIF), and closed reduction and internal fixation (CRIF), with different fixation options.

Femoral neck fractures are associated with multiple postoperative complications, including superficial or deep infections, malunion (where the fractured bone heals in an abnormal position) or nonunion (where the bones fail to heal), fixation failure, venous thromboembolism (VTE), death, and avascular necrosis (AVN) due to disruption of arterial blood flow, among other complications [8]. Previous studies have researched the relationship between different variables and the possible complications that may follow. A retrospective data analysis of 552 cases treated with hemiarthroplasty between January 2017 and December 2023 investigated multiple factors that influenced the complication rate post HA and identified surgical duration as a significant factor influencing the likelihood of postoperative complications [9]. Additionally, a multicentre study outlining factors associated with FNF complications showed that the FNF complications were linked to the injury mechanism and the type of fixation [10]. A retrospective analysis of cases between 2009 and 2019 identified several risk factors associated with postoperative complications. Cerebrovascular disease, concomitant fractures, and rheumatoid disease were identified in patients who subsequently developed union failure. The use of anticoagulant therapy was observed among patients who experienced fracture-related infections. Chronic obstructive pulmonary disease was observed in patients who later developed mechanical complications [11]. The Kaplan-Meier (KM) method was used in another retrospective analysis to determine the mortality rate in proximal femur fracture cases. Results estimated the mortality rate: 26.8% in 1 year following head/neck fracture, 28.2% following intertrochanteric fracture, and 24.2% following subtrochanteric fracture. The results also identified many factors that influence the mortality rate, including male sex, age more than 70, diabetes mellitus, hypertension, and insulin and tobacco use [11].

Despite the published articles about FNF complications, no similar study has been published in Saudi Arabia, highlighting a gap in localized research. This study aimed to identify clinical, fracture-related, and treatment-related predictors of postoperative complications and one-year mortality among patients with FNFs using multivariate logistic regression analysis at King Abdulaziz Medical City, by analyzing patients' records from January 2016 to August 2024, to provide insight that may improve clinical outcomes.

## Materials and methods

A retrospective study was conducted using the database of King Abdulaziz Medical City, Jeddah, Saudi Arabia, from 2016 to 2024. All adult patients admitted with radiologically confirmed FNFs between January 2016 and August 2024 were included. Patients with incomplete medical records or missing key variables required for analysis were excluded.

Clinical and demographic variables were retrieved from electronic medical records. These included age, sex, body mass index (BMI), comorbidities (hypertension, diabetes mellitus, chronic kidney disease, osteoporosis, osteopenia, dyslipidemia), and fracture characteristics (basicervical, subcapital, transcervical; the Garden classification). The Garden classification was determined based on preoperative hip radiographs as documented in the orthopedic and radiology records. Treatment variables included surgical versus conservative management, type of surgery (hemiarthroplasty, total hip arthroplasty (THA), open reduction and internal fixation (ORIF), closed reduction and internal fixation (CRIF)), and time to surgery. Time to surgery was defined as the interval between hospital admission and the time of surgical intervention. Outcomes of interest were hospital length of stay, postoperative complications, and all-cause mortality within one year. One-year mortality was verified using hospital electronic medical records and documented follow-up information. Complications included infection, thromboembolic events, fixation failure, refracture, malunion or nonunion, and avascular necrosis. Postoperative infection included both superficial and deep surgical site infections documented in the medical records. Thromboembolic events included deep vein thrombosis (DVT) and pulmonary embolism (PE) confirmed by imaging or physician documentation.

The research protocol underwent ethical review and received formal approval from Abdullah International Medical Research Center (KAIMRC) (#0000064024). Given the retrospective design, informed consent was waived. All data were anonymized before analysis to ensure confidentiality.

Data were analyzed using IBM SPSS Statistics, version 29.0 (IBM Corp., Armonk, NY, US). Continuous variables were tested for normality using the Shapiro-Wilk test. Normally distributed variables were presented as mean ± standard deviation (SD), while skewed variables were reported as medians with interquartile ranges (IQR). Categorical variables were expressed as frequencies and percentages. Comparisons between groups (mortality vs. survival; complications vs. no complications) were made using the student’s t-test or Mann-Whitney U test for continuous variables and Chi-square or Fisher’s exact tests for categorical variables.

Univariate analysis was initially performed to assess associations between clinical variables and outcomes. Variables with p < 0.05 on univariate analysis were entered into a multivariate logistic regression model to identify independent predictors of mortality and complications. Results were reported as odds ratios (OR) with 95% confidence intervals (CI). A p-value of < 0.05 was considered statistically significant.

## Results

The study included 183 patients with FNFs, with a mean age of 71.8 ± 17.0 years and a mean BMI of 26.3 ± 6.1 kg/m². The mean hospital stay was 10.7 ± 8.6 days. Females comprised 104 (56.8%) of the cohort. Fracture types included basicervical (43; 23.5%), subcapital (61; 33.3%), and transcervical (79; 43.2%) fractures. According to the Garden classification, 53 (29.0%) were Grade II, 106 (57.9%) Grade III, and 24 (13.1%) Grade IV. The most prevalent comorbidities were hypertension (107; 58.5%), diabetes mellitus (85; 46.4%), osteopenia (70; 38.3%), osteoporosis (48; 26.2%), CKD (32; 17.5%), and dyslipidemia (32; 17.5%). Surgical treatment was performed in 171 (93.4%) patients, most commonly hemiarthroplasty (113; 61.7%), followed by closed reduction internal fixation (CRIF) (26; 14.2%), open reduction and internal fixation (ORIF) (25; 13.7%), and total hip arthroplasty (THA) (7; 3.8%). Conservative management was used in 12 (6.6%) cases and was associated with a 25% mortality rate (3/12). While overall complications occurred in 48 (26.2%) patients and 13 (7.1%) died within one year, the mortality risk among surgically treated patients was notably lower at 5.85% (10/171). Table 1 shows the baseline characteristics of the study population. 

**Table 1:** Baseline characteristics of the study population (n = 183) Data are mean ± SD or n (%)

Variable	n (%) or Mean ± SD
Age, years (mean ± SD)	71.8 ± 17.0
BMI, kg/m² (mean ± SD)	26.3 ± 6.1
Length of stay, days (mean ± SD)	10.7 ± 8.6
Gender	
Female	104 (56.8%)
Male	79 (43.2%)
Type of fracture	
Basicervical	43 (23.5%)
Subcapital	61 (33.3%)
Transcervical	79 (43.2%)
Garden classification	
Grade II	53 (29.0%)
Grade III	106 (57.9%)
Grade IV	24 (13.1%)
Comorbidities ( n of patients )	
Osteopenia	70 (38.3%)
Osteoporosis	48 (26.2%)
Hypertension	107 (58.5%)
Diabetes mellitus	85 (46.4%)
Chronic kidney disease (CKD)	32 (17.5%)
Dyslipidemia	32 (17.5%)
Type of treatment	
Surgical treatment	171 (93.4%)
Conservative treatment	12 (6.6%)
Type of surgery (n=171)	
Hemiarthroplasty	113 (61.7%)
Closed reduction internal fixation* *(CRIF)	26 (14.2%)
Open reduction and internal fixation (ORIF)	25 (13.7%)
Total hip arthroplasty (THA)	7 (3.8%)
Complications (any)	48 (26.2%)
Mortality	13 (7.1%)

When factors associated with mortality were examined, Garden classification (p = 0.011), CKD (p = 0.039), type of treatment (p = 0.013), and infection (p = 0.042) showed statistically significant associations in univariate analysis. No significant differences were found in age, BMI, gender, fracture type, hypertension, diabetes mellitus, osteopenia, osteoporosis, or dyslipidemia between survivors and non-survivors. The mean age for survivors and non-survivors was 72.2 ± 16.6 years and 71.0 ± 18.4 years, respectively (p = 0.676). Table 2 lists the factors associated with mortality.

**Table 2:** Factors associated with mortality *Indicates statistically significant p-value (p < 0.05) A hyphen (-) indicates not applicable.

Variable	No Death (n = 170)	Death (n = 13)	p-value
Age (years), mean ± SD	72.2 ± 16.6	71.0 ± 18.4	0.676
BMI, kg/m² (mean ± SD)	26.3 ± 6.2	25.0 ± 4.4	0.468
Length of stay, days (mean ± SD)	10.5 ± 8.4	12.2 ± 10.4	0.514
Gender			0.165
Female	99 (58.2%)	5 (38.5%)	-
Male	71 (41.8%)	8 (61.5%)	-
Type of fracture			0.972
Basicervical	40 (23.5%)	3 (23.1%)	-
Subcapital	57 (33.5%)	4 (30.8%)	-
Transcervical	73 (42.9%)	6 (46.2%)	-
Garden classification			0.011*
Grade II	52 (30.6%)	1 (7.7%)	-
Grade III	99 (58.2%)	7 (53.8%)	-
Grade IV	19 (11.2%)	5 (38.5%)	-
Time to surgery (n=171)			0.725
<24 hours	35 (21.7%)	2 (20%)	-
<48 hours	55 (34.2 %)	3 (30%)	-
<72 hours	29 (18%)	1 (10%)	-
<7 days	32 (19.2%)	4(40%)	-
<14 days	7 (4.3%)	0	-
>14 days	3 (1.9%)	0	-
Comorbidities ( n of patients )			
Osteopenia	66 (38.8%)	4 (30.8%)	0.543
Osteoporosis	45 (26.5%)	3 (23.1%)	0.699
Hypertension	100 (58.8%)	7 (53.8%)	0.726
Diabetes mellitus	79 (46.5%)	6 (46.2%)	0.579
Chronic kidney disease (CKD)	26 (15.3%)	6 (46.2%)	0.039*
Dyslipidemia	29 (17.1%)	3 (23.1%)	0.335
Type of treatment			0.013*
Surgical treatment	161 (94.7%)	10 (76.9%)	-
Conservative treatment	9 (5.3%)	3 (23.1%)	-
Infection	11 (6.5%)	4 (30.8%)	0.042*

Analysis of predictors for complications revealed that patients who developed complications had a significantly longer mean hospital stay (13.5 ± 12.8 days) compared to those without complications (9.6 ± 6.2 days, p = 0.007). No statistically significant associations were observed between complications and age, BMI, gender, or comorbidities. Although higher proportions of osteoporosis, hypertension, and osteopenia were noted among patients with complications, these differences did not reach statistical significance. Table 3 lists the predictors of complications.

**Table 3:** Predictors of complications *Indicates statistically significant p-value (p < 0.05) A hyphen (-) indicates not applicable.

Variable	No Complications (n = 135)	Complications (n = 48)	p-value
Age (years), mean ± SD	71.5 ± 17.3	76.9 ± 12.4	0.265
BMI (kg/m²), mean ± SD	26.6 ± 6.1	25.6 ± 6.1	0.233
Length of stay, days (mean ± SD)	9.6 ± 6.2	13.5 ± 12.8	0.007*
Time to surgery (n=171)			0.448
<24 hours	30 (23.4%)	7 (16.3%)	-
<48 hours	41 (32%)	17 (39.5%)	-
<72 hours	22 (17.2%)	8 (18.6%)	-
<7 days	29 (22.7%)	7 (16.3%)	-
<14 days	5 (3.9%)	2 (4.7%)	-
>14 days	1 (0.8%)	2 (4.7%)	-
Gender			0.147
Female	81 (60.0%)	23 (47.9%)	-
Male	54 (40.0%)	25 (52.1%)	-
Comorbidities ( n of patients )			
Osteopenia	43 (31.9%)	27 (56.3%)	0.901
Osteoporosis	32 (23.7%)	16 (33.3%)	0.092
Hypertension	74 (54.8%)	33 (68.8%)	0.084
Diabetes mellitus	61 (45.2%)	24 (50.0%)	0.439
Chronic kidney disease (CKD)	23 (17.0%)	9 (18.8%)	0.788
Dyslipidemia	29 (17.1%)	3 (23.1%)	0.290

Multivariate logistic regression identified the Garden classification as an independent predictor of mortality (OR 3.09, 95% CI 1.10-8.67, p = 0.032). CKD (OR 3.68, p = 0.052), infection (OR 4.38, p = 0.079), and type of treatment (OR 3.84, p = 0.104) showed a trend toward significance but did not reach statistical significance. Table 4 shows the multivariate logistic regression for mortality.

**Table 4:** Multivariate Logistic Regression for Mortality *Indicates statistically significant p-value (p < 0.05)

Variable	OR (Exp(B))	95% CI for OR	p-value
Chronic kidney disease (CKD)	3.68	0.99 – 13.69	0.052
Infection	4.38	0.84 – 22.76	0.079
Garden classification	3.09	1.10 – 8.67	0.032*
Type of treatment	3.84	0.76 – 19.45	0.104

## Discussion

This study included 183 patients with femoral neck fractures, most of whom underwent surgical treatment, with hemiarthroplasty being the most common intervention. The overall 1-year mortality rate was 7.1%. The relatively low mortality rate in our cohort, compared to a study by Walter et al., which reported 26.8% mortality after a femoral head/neck fracture at one year, may reflect improvements in perioperative care [11]. Moreover, we found that the Garden classification is an independent predictor of mortality, aligning with findings from the Swedish Fracture Register, in which displaced FNFs (Garden 3 and 4) had a higher mortality rate than those with nondisplaced fractures (Garden 1 and 2) [12]. These results highlight the crucial role of fracture classification in not only guiding surgical decision-making but also in predicting survival outcomes.

In addition to fracture severity, patient comorbidities and perioperative complications were significant determinants of mortality. In our study, CKD was significantly associated with increased risk of death. This is consistent with prior evidence showing that renal impairment adversely affects outcomes after hip fractures due to altered bone metabolism [13]. Similarly, postoperative infections were strongly linked to higher mortality in our cohort. Roche et al. reported that medical complications, such as wound infection, substantially increased both short- and long-term mortality after hip fracture surgery [14]. Treatment modality also influenced survival. Although hemiarthroplasty was the most common procedure in our series, patients managed conservatively demonstrated a significantly higher mortality rate (25%, 3/12) compared to those treated surgically (5.85%, 10/171). This reflects the vulnerability of nonoperative candidates. Rogmark and Leonardsson found that arthroplasty generally provides better long-term survival compared with nonoperative care in elderly patients with displaced femoral neck fractures [5]. However, the literature suggests that the choice of fixation versus arthroplasty must be made after assessing the fracture type, comorbidities, and functional status [1]. Taken together, these findings show the importance of a multifactorial approach in reducing mortality risk, performing early surgery, optimizing comorbidities, and aggressively preventing perioperative infections.

Regarding complications, our study revealed that patients who had one or more complications had a significantly longer length of hospital stay. Similarly, a recent study from New South Wales found that older adults with hospital-acquired complications after a hip fracture had a median hospital stay of 31 days compared to 22 days for those without complications [15]. The complication rate in our cohort (26.2%) was comparable to findings from Saudi Arabia, where Al-Mohrej et al. reported a 32.2% complication rate following hip arthroplasty [16, and aligned with international reports citing 25-35% complication rates [17]. Although comorbidities such as osteoporosis, hypertension, and diabetes were more common in patients with complications, these associations were not statistically significant.

This study has some limitations that should be acknowledged. Its retrospective and single-center design may limit the generalizability of the findings, and unmeasured confounders, such as nutritional status and socioeconomic factors, were not assessed. The relatively small number of mortality events in our cohort may have reduced the statistical precision of the regression estimates and resulted in wider confidence intervals. In addition, some potentially relevant clinical variables, such as pre-fracture functional status, cognitive impairment, and American Society of Anesthesiologists (ASA) classification, could not be included in the analysis.

Even with these limitations, our study carries important strengths. We were able to include a relatively large number of patients compared with other studies from the region, and we provided a broad look at clinical, surgical, and complication-related outcomes. We also placed our findings in context by comparing them with both regional and international reports. Future research would benefit from multicenter prospective studies that use standardized measures to assess the patients’ functional recovery.

## Conclusions

In this cohort of adult patients with femoral neck fractures, fracture displacement, as assessed by the Garden classification, independently predicted one-year mortality. Postoperative complications were associated with significantly prolonged hospital stay but were not independently associated with mortality after adjustment. Chronic kidney disease, postoperative infection, and conservative management were associated with mortality on univariate analysis only. These findings emphasize the prognostic role of fracture severity and the clinical impact of postoperative complications on hospitalization.
